# Supplementary material for: DeepPL: A deep-learning-based tool for the prediction of bacteriophage lifecycle
Source: PLoS Comput Biol. 2024 Oct 17;20(10):e1012525. doi: 10.1371/journal.pcbi.1012525 (PMC11521287; doi:10.1371/journal.pcbi.1012525)
Supplement: Attachment — Submitted filename: review.pdf [file pcbi.1012525.s004.pdf]

This work leverages the pre-trained NLP model DNABERT to predict the life cycle of phages based on their genome sequences. The model achieved impressive performance and the in-silico work was well experimentally validated. While the work has several strengths, there are a few conceptual and structural ambiguity. The author well emphasizes the importance of this work, but their emphasis on their previous work PhaTYP can be appropriately reduced. Some important details are not well interpreted in the results part of this paper but mentioned in the figures and methods, which cause confusion to readers at the beginning. The authors need to work on the narrative framework and the language. So far, the incremental nature of this work is not perfectly developed, and further analysis would refine this work.

General remarks:

Results - Performance assessment

It is more natural to introduce the framework of the model in this section first and then following with the performance assessment. Methods and figure 1 mentioned the feature extraction, but I would suggest explaining it in detail in this section. It was very confusing what are the features and why the author chose them in the current submission. Moreover, the thresholds 1 and 2 are not well-explained in the main part of manuscript, and this will cause ambiguity for the readers. And a few questions about the model DeepPL: 1) DNABERT is pre-trained on human genomes and in their paper, they only generalized it to other mammalian organisms. The genomes of bacterial phages are relatively different from human genomes, more rationales are needed to apply DNABERT in this scenario. 2) For the model performance metrices, it is more common to use AUC/AUROC and F1 score than accuracy.

Results - Case study 1-In-house verified phages

Some narrative ambiguity: 19 genomes from the phages isolated by our lab – Are those 19 different strains of phages? Are those phages were predicted based on the complete genome sequencing? Phage Sa179lwIf has the questionable lifecycle – I wonder what is the prediction of DeepPL for this strain of phage? How do you interpret the prediction results in this case? Does this indicate the ground truth of phage lifecycle is not super reliable?

Results - Case study 2-Metagenomic data

How did the author generate the mock phage metagenomic data is a little bit confusing. More specifically, how did the author convert complete genomes of phages to metagenomic contigs? What percentage of the complete genomes are represented by

the simulated metagenomic contigs of each strain? Which parts of the phage genomes are covered in the metagenomic contigs? In the real scenario, is it ensured to capture the selected features like the lysogenic genes mentioned above? Plus, comparison to other tools in this section would be natural.

Fig1.

Less emphasis on the data collection, not too much useful information for data collection step in the current version of figure.

The feature engineering/extraction step was not well explained in the main part of the manuscript but was emphasized in fig 1. Need to explain it in the results.

12X in fig1 was ambiguous. Add some annotation to that.

Better to describe the threshold 1 and 2 in the fig 1 as well. In my understanding, there are 2 steps of classification: step 1 is to find if there is a lysogenic gene in the input sequence; step 2 is a final prediction of phage lifecycles. How does the author integrate the prediction of step 1 to step 2 is ambiguous.

Fig2.

The 2 colors are too similar. A more muted color palette is recommended. And it is more natural to also compare to other similar tools in this figure.

More analysis of the explainability of DeepPL

Since the authors manually selected lysogenic genes as the input for the model instead of using the whole phage genomes as input, it would be great if the pattern or motif can be identified from the DNA sequences. Then using the found pattern to enhance the explianability of this model will further refine this work given the fact that DNABERT is multihead attention model and it will emphasize on certain part of the input sequences.
