# Supplementary material for: DeepPL: A deep-learning-based tool for the prediction of bacteriophage lifecycle
Source: PLoS Comput Biol. 2024 Oct 17;20(10):e1012525. doi: 10.1371/journal.pcbi.1012525 (PMC11521287; doi:10.1371/journal.pcbi.1012525)
Supplement: Attachment — Submitted filename: Response letter to reviewers submitted.pdf [file pcbi.1012525.s005.pdf]

Response Letter to the Reviewer's Comments

Manuscript ID: PCOMPBIOL-D-24-00537

Manuscript Title: DeepPL: a deep-learning-based tool for the prediction of bacteriophage lifecycle

Author(s): Yujie Zhang, Mark Mao, Robert Zhang, Yen-Te Liao, and Vivian C.H. Wu

Reviewer #1:

The authors introduce a deep learning tool based on natural language processing techniques to predict bacteriophage lifestyles from nucleotide sequences (DeepPL). This tool uses the DNABERT model, a variant of the popular BERT model adapted for DNA sequences, and demonstrates an accuracy of 94.65%, with sensitivity and specificity rates of 92.24% and 95.91% respectively. DeepPL performs comparably to existing tools and has shown promising results in both lab-verified phage genomes and metagenomic datasets, achieving 100% accuracy on individual phage genomes and varying high accuracies on phage contigs. However, I have several concerns about this work:

1. Figure 1 Clarification: Figure 1 is difficult to interpret. For instance, in the Feature Extraction panel, it's unclear what the annotations P, O, Q, CI represent-are these proteins? Also, does DeepPL require protein inputs? What does the highlighted ACGT sequence signify? In the Model Training panel, what does "12 X" mean? Is it indicating that the input K-mer vectors are processed through 12 Transformer blocks? Are the pre-training and fine-tuning steps using the same Transformer block structures? He, et al [1] mention the use of residual connections to prevent overfitting. It would be beneficial to include it in the Transformer block layer normalization.

Response: We appreciate the reviewer's comment and have updated Figure 1 in the revised manuscript. In the updated Figure 1, the lysogenic genes (P, O, Q, CI etc) have been labeled with nucleotide sequence (ATGC sequence) to indicate that they are genes, not proteins. The input of DeepPL only needs nucleotide sequences instead of protein sequence. 12X refers to 12 transformer blocks and is indicated in Figure 1. DNABERT was pre-trained using the same model architecture as the BERT base, which consists of 12 Transformer blocks. Therefore, we further conducted fine-tuning using the same transformer block structures. During the fine-tuning process, we created a callback function to stop the training if it detects the overfitting. The evaluation results of our fine-tuning process further validated that there was no overfitting for the model.

2. Dataset Description: It would be beneficial to include a brief description of the datasets in the Results section for better clarify and context.

Response: We appreciate the reviewer's comment and have included a brief description of the dataset in the revised manuscript (lines 184-189). In addition, we have moved the method section before the results section for a better manuscript structure.

3. Performance Assessment: The rationale behind using a balanced training set in the 5-fold cross-validation should be explained. Additionally, Table 1 seems redundant and could potentially be omitted for brevity.

Response: We appreciate the reviewer's comment and have updated the descriptions of the balanced training dataset in the revised manuscript (lines 184-189). Table 1 has been changed to supplementary data 2 to provide extra information regarding the model performance stability of DeepPL.

4. Method Comparison: The comparison with previously published methods should include a ROC curve to better illustrate the trade-offs between sensitivity and specificity. Also, it would be good if author could explain the low sensitivity observed with PHACTs. The running time comparison of different methods should be included.

Response: We appreciate the reviewer's comment and have updated the ROC curve in the revised manuscript (lines 205-208, Figure 2). The training dataset of PHACTs consisted of 600 proteins generated from 148 temperate phages and 79 virulent phages. However, as mentioned in the introduction and method sections, temperate phage can display both lytic and lysogenic cycles whereas virulent phage can only display lytic cycles. The key genetic difference between these two phage types is the lysogenic genes within the temperate phage genomes (around 8% within the phage complete genome). The randomly selected protein sequences of PHACTs training dataset likely included a large portion of proteins both presented in temperate and virulence phages. Therefore, the performance of PHACTs showed low sensitivity when temperate phages were referred to as positive samples. The running time of five tools included in this study can't be compared due to the different installation and computing environment settings. For example, PhageAI can only do the phage lifecycle prediction in their webserver. Therefore, we provide the running time of DeepPL under different environments (M1 and Nvidia V100) in the manuscript for reference (lines 327-330).

5. Case Study 1: Both PhaTYP and DeepPL predict with 100% accuracy for the lifestyle of 18 phages, yet one questionable phage, Sa1791w, presents a controversial lifestyle, making the conclusion that PhaTYP has lower accuracy than DeepPL. The conclusion is unconvincing.

Response: We appreciate the reviewer's comment. In case study 1, we mainly focus on validating DeepPL prediction results using in-house verified phages. We also involved the phage Sa179lw to highlight the high sensitivity of DeepPL. As we mentioned in the discussion (lines 301-307), due to the risk of lysogenic potential, the virulent phages carrying lysogenic modules will not be considered for phage therapy or biocontrol purposes. Therefore, we trade-offs between accuracy and sensitivity to make DeepPL more applicable in reality.

6. Case Study 2: Clarify what "blastn" refers to in the text. Additionally, explain the presence of several NAs in Table 4 and ensure consistency in the formatting of the 'Sequence length (bp)'

column across tables. The sequencing platform and assembly method should also be included. For Figure 2, include the sample size for each bar and consider using blue/red for better visual distinction as complementary colors.

Response: We appreciate the reviewer's comment and have updated the full name of blastn in the revised manuscript. NA refers to the information is not available and is indicated in the footnote of Table 3 (previous Table 4). The tables have been updated for consistency in format. The sequencing platform and assembly method was included in the line 248-249. Figure 3 (previous Fig 2) with complementary colors and sample size has also been updated in the revised manuscript.

7. Data Collection Consistency: The authors state that representative lysogenic gene markers were selected, but performance on uncoded genes or a mix (partial coded) would be interesting to see. Otherwise, I feel like no difference between PhaTYP and DeepPL. Discrepancies in the number of lytic and lysogenic samples between the manuscript and Supplementary Data 2 should be addressed.

Response: We appreciate the reviewer's comment. The test datasets are all nucleotide sequences (uncoded sequences). In addition, to test the partial or complete phage sequences, we conducted Case study 1 (complete phage nucleotide sequences) and Case study 2 (complete phage nucleotide sequences and partial phage nucleotide sequences) to test the performance of DeepPL. The prediction of phage lifecycle using complete phage genomes is a relatively easy task, compared to the partial sequence, which lost a lot of lysogenic features. In case study 2, DeepPL still can recognize the lysogenic features with limited information compared to the PhaTYP. The discrepancies in the number of lytic and lysogenic samples involved in this manuscript have been indicated in lines 130-133 that more virulent phages with biological verification have been reported in the National Center for Biotechnology Information (NCBI) database compared to temperate phages.

8. K-mer, sliding window and Learning Rate: Clarify what "k-mer 6 sequences" refers to – is it sequences of six nucleotides? The decision to use a skip step for a balanced training dataset, use 100 bp in sliding window and the relationship between learning rate and epoch should be elaborated on.

Response: We appreciate the reviewer's comment. Kmer 6 refers to the nucleotide sequence of six nucleotides (i.e., ATACAG TACAGC ACAGCT CAGCTT AGCTTT). The optimization of the skip steps for the lysogenic gene dataset and lytic phage genome has been conducted to achieve a 50% -50% balanced training dataset. For example, group 1 has 226,625 DNA sequences of lysogenic genes and 229,055 DNA sequences of lytic phage genomes. In the script (batch_gen_kmer6_from_fasta_files.py) we provided in GitHub, it can adjust the skip step for each category and print the lines of lysogenic and lytic DNA sequences to calculate and have a balanced training dataset. We also tested the sliding window with different sizes (100, 200, 300, 400, and 512 bp). It showed no significant difference of AUC between these sizes (around 0.86 for each size, group1234 as the training dataset and group5 as the test dataset). Therefore, we went for the sliding window of 100bp to minimize the requirement of computing memory. The

learning rate determines the step size of each update during training, while epochs determine how many times the entire dataset is used for training. Therefore, we used a larger learning rate in early epochs. To prevent the risks of overshooting the optimal solution, further, we used a smaller learning rate in later epochs to achieve good performance, as the model makes smaller adjustments with each update.

9. Minor. At line 111, could you please clarify whether you are referring to the conversion from amino acid sequences to nucleotide sequences?

Response: We appreciate the reviewer's comment and have updated the revised manuscript. We are referring to the conversion from nucleotide sequence to amino acid sequence (line 109).

10. Minor. In the discussion section, consider including references [2, 3], which discuss the use of phylogenetic tree information (highlighting greater similarity among closely related entities) to improve the predictive power and association test power.

Response: We appreciate the reviewer's comment and have included the prediction power of the phylogenetic tree in the revised manuscript (lines 332-335).

[1] He K, Zhang X, Ren S, et al. Deep residual learning for image recognition. In: Proceedings of the IEEE Conference on Computer Vision and Pattern Recognition, Manhattan, New York, NY, United Stated: Institute of Electrical and Electronics Engineers, 2016, 770–8.
[2] Walkup, J., Dang, C., Mau, R.L. et al. The predictive power of phylogeny on growth rates in soil bacterial communities. ISME COMMUN. 3, 73 (2023). https://doi.org/10.1038/s43705-023-00281-1
[3] Hong, Q., Chen, G. & Tang, ZZ. PhyloMed: a phylogeny-based test of mediation effect in microbiome. Genome Biol 24, 72 (2023). https://doi.org/10.1186/s13059-023-02902-3

Reviewer #2:

The authors present a deep-learning tool (DeepPL) based on the previously published DNABERT (Bidirectional Encoder Representations from Transformers for DNA language) model. The goal of DeepPL is to classify phage genomes as either lytic or lysogenic based on their sequence. DeepPL uses the pre-trained DNABERT model and fine-tunes it for the new task in the space of Bacteriophages. The authors demonstrate that their tool performs favorably to four other methods. It is an interesting problem and as we need to improve our understanding of the interactions between viruses and bacteria, I think this work is timely. Overall, I find the paper to be well written, however, I have two major concerns about the way key parameters were selected as discussed below.

Major remarks:

Line 300 "The comparison of different parameters indicated that k mer 6 and sequence size of

100 bp yielded reasonably balanced results and were further used in the current study. "The comparison is nowhere to be found in the manuscript and no description is given as to what a balanced result is

Response: We appreciate the reviewer's comment and have updated the revised manuscript (lines 151-157). Kmer 6 was selected based on the results of DNABERT. The sliding window with different sizes (100, 200, 300, 400, and 512 bp) was tested and showed there was no significant difference in AUC between these sizes (around 0.86 for each size, group1234 as the training dataset and group5 as test dataset). Moreover, the sliding windows of 100 bp only require 10GB of memory NVidia a100, while sliding windows of 300 bp or above requested 60GB of memory of NVidia a100 for the fine-tuning process. Therefore, we went for the sliding window of 100bp to minimize the requirement of computing memory.

How did you decide on the size of the skip step for lytic phage genomes? What tests were done to justify a much larger step size of 91? Why should different skip-steps be used to begin with (you can balance the sets at k-mer level rather than gene level)? When you use skip-step=1, you will have many overlapping k-mers, with skip-step=91, none at all. How would this affect the model training and performance? I found the lack of any evidence and discussion to support the choice of (all) your parameters astonishing

Response: We appreciate the reviewer's comment and have updated the description in the revised manuscript (lines 151-157). For the DNABERT fine-tuning process, we can only set the same kmer for two phage types. In addition, DNABERT tested different kmers and kmer6 achieved the best performance. Therefore, we adjusted the skip step instead of different kmers for two categories. We further developed the script (batch_gen_kmer6_from_fasta_files.py) provided in the github) to generate a balanced dataset. This script can adjust the skipped step for each category and print the lines of lysogenic and lytic DNA sequences to calculate and have a balanced training dataset. After the test of different skip steps, the skip step of 1 and 91 for lysogenic gene dataset and lytic phage genome were selected in this study to achieve a 50% - 50% balanced training dataset (547,810 DNA sequences from the lytic phage genome and 500,765 DNA sequences from lysogenic genes). BERT requests a balanced dataset to achieve the best performance, meaning that all characteristic patterns of each class have roughly equal chances in model training progress. The classification of two phage types will likely result in similar accuracy. If there is a class imbalance, the algorithm may have trouble learning to classify the underrepresented class, and this can lead to poor performance in that class. Therefore, compared to the overlapping input, the full coverage and the balanced dataset of two different phage-type sequences are highly associated with the BERT performance. Due to the imbalanced number of temperate and virulent phage genomes in the NCBI database, we decided to balance the training dataset using different skip steps.

You report results based on five fold cross-validation. Yet, in the Data Collection section (line 283) it is stated that "additional 374 complete phage genomes with reliable lifecycle labels were collected from the NCBI database as the test dataset." So, was it a 5-fold cross-validation or an external testset?

Response: We appreciate the reviewer's comment and have updated a description in the revised manuscript (184-190). The five-fold cross-validation was conducted by randomly dividing the training dataset into five equal-sized groups. Different from the training dataset, the test dataset is additional 374 complete phage genomes with reliable lifecycle labels collected from the NCBI database.

Minor remarks:

It's unclear in the caption of Figure 1 what threshold 1 is as it is not in the figure.

Response: We appreciate the reviewer's comment and have updated the thresholds 1 and 2 in the updated Figure 1.

How was the aggregation of frames done to obtain a score for threshold2?

Response: We appreciate the reviewer's comment. The script (batch_process_results.py) provided in the GitHub provided the function of adjusting the setting of threshold 2 and generating the error rate of test dataset. By using this script, we tested different settings of threshold 2 and further selected the threhold2 of 0.016, which can yield reasonably balanced results.

Line 281: "Therefore, a total of 1,262 virulent phage genomes with the strictly lytic cycle were verified and used to balance the training dataset." - expand to make clear you balance the number of genes.

Response: We appreciate the reviewer's comment and have updated the numbers of DNA sequences in the revised manuscript (lines 159-160). The different skip steps for the lysogenic gene dataset and lytic phage genome have been conducted to achieve a 50% -50% balanced training dataset. In detail, the training dataset included 547,810 DNA sequences from the lytic phage genome and 500,765 DNA sequences from lysogenic genes.

Fig2: colors are hard to distinguish

Response: We appreciate the reviewer's comment and have updated Figure 3 (previous Figure 2) in the revised manuscript.

Table3: mark in bold the best performer in each category

Response: We appreciate the reviewer's comment and have updated the tables in the revised manuscript.

Table4: hard to read as column 1 takes two or there rows

Response: We appreciate the reviewer's comment. We uploaded the pdf file of our updated manuscript to make it easy to read.

Reviewer #3:

This work leverages the pre-trained NLP model DNABERT to predict the life cycle of phages based on their genome sequences. The model achieved impressive performance and the in-silico work was well experimentally validated. While the work has several strengths, there are a few conceptual and structural ambiguity. The author well emphasizes the importance of this work, but their emphasis on their previous work PhaTYP can be appropriately reduced. Some important details are not well interpreted in the results part of this paper but mentioned in the figures and methods, which cause confusion to readers at the beginning. The authors need to work on the narrative framework and the language. So far, the incremental nature of this work is not perfectly developed, and further analysis would refine this work.

Response: We appreciate the reviewer's comment and have moved the methods section before the results section for a better manuscript structure

General remarks:

Results - Performance assessment

It is more natural to introduce the framework of the model in this section first and then following with the performance assessment. Methods and figure 1 mentioned the feature extraction, but I would suggest explaining it in detail in this section. It was very confusing what are the features and why the author chose them in the current submission. Moreover, the thresholds 1 and 2 are not well-explained in the main part of manuscript, and this will cause ambiguity for the readers. And a few questions about the model DeepPL: 1) DNABERT is pre-trained on human genomes and in their paper, they only generalized it to other mammalian organisms. The genomes of bacterial phages are relatively different from human genomes, more rationales are needed to apply DNABERT in this scenario. 2) For the model performance metrices, it is more common to use AUC/AUROC and F1 score than accuracy.

Response: We appreciate the reviewer's comment and have moved the materials and method before the results section for a better manuscript structure. The explanation of thresholds 1 and 2 have been provided in the method section (lines 170-175) and updated in Fig 1. Although DNABERT is pre-trained on mammalian genomes, one important feature of BERT is that fine-tuning with a relatively small specific training set can yield great results for new downstream tasks. Taking advantage of DNABERT with trained knowledge of genome nucleotide sequences and the feature of the unified architecture across various downstream tasks demonstrated its potential ability in the bacteriophage DNA language. Our DeepPL performance further validated this hypothesis. In addition, the ROC curve has been updated in the revised manuscript. The F1 score was provided in the Supplementary data 2 (previous Table 1).

Results - Case study 1-In-house verified phages

Some narrative ambiguity: 19 genomes from the phages isolated by our lab – Are those 19 different strains of phages? Are those phages were predicted based on the complete genome sequencing? Phage Sa179lwIf has the questionable lifecycle – I wonder what is the prediction of DeepPL for this strain of phage? How do you interpret the prediction results in this case? Does this indicate the ground truth of phage lifecycle is not super reliable?

Response: We appreciate the reviewer's comment. The 19 different phages were different strains and isolated from different sources. The prediction of these phages was based on the phage's complete nucleotide sequences. The prediction of Sa179lw was predicted as lysogenic phage and indicated in Table 2 (previous Table 3). In case study 1, we involved the phage Sa179lw to highlight the high sensitivity of DeepPL which can detect the presence of lysogenic modules. As we mentioned in the discussion (lines 301-307), due to the risk of lysogenic potential, the virulent phages carrying lysogenic modules will not be considered for phage therapy or biocontrol purposes. Therefore, we trade-offs between accuracy and sensitivity to make DeepPL more applicable.

Results - Case study 2-Metagenomic data

How did the author generate the mock phage metagenomic data is a little bit confusing. More specifically, how did the author convert complete genomes of phages to metagenomic contigs? What percentage of the complete genomes are represented by the simulated metagenomic contigs of each strain? Which parts of the phage genomes are covered in the metagenomic contigs? In the real scenario, is it ensured to capture the selected features like the lysogenic genes mentioned above? Plus, comparison to other tools in this section would be natural.

Response: We appreciate the reviewer's comment. The mock phage metagenomic data was generated by the study conducted by Dr. Cook (line 237, reference 24: Cook R, Brown N, Rihtman B, et al. The long and short of it: Benchmarking viromics using Illumina, Nanopore and PacBio sequencing technologies. Microbial Genomics 2024, 10.2:001198.). Detailed information regarding the phage complete genome and metagenomic sequencing were provided the reference 24. Briefly, the authors mixed the 15 phages' DNA and conducted the metagenomic sequencing using different sequencers, which were further assembled using different bioinformatic tools. Therefore, no specific parts of the phage genomes, such as lysogenic genes, were covered in the metagenomic contigs. Some metagenomic contigs contain limited/none lysogenic genes. Our study selected this dataset for the following reasons. 1) These 15 phages had complete genomes, and most of their lifecycles were confirmed by biological experiments. Therefore, this information can help us validate the prediction of metagenomic contigs. 2) This metagenomic dataset included the most common sequencer and bioinformatic tools used for the viral metagenomic study. One challenge of viral metagenomics, phageome in particular, is that it is hard to identify phage lifecycle via short metagenomic contigs. The prediction of phage lifecycle using complete phage genomes is a relatively easy task compared to the partial sequence (metagenomic contigs), which lost a lot of lysogenic features. Therefore, we would like to test the performance of DeepPL on this dataset to evaluate its performance on viral metagenomic contigs. Based on the performance of five tools on the test dataset of 374 phage complete genomes, we decided only to test DeepPL and PhaTYP for two case studies.

Fig1.

Less emphasis on the data collection, not too much useful information for data collection step in the current version of figure.

Response: We appreciate the reviewer's comment. In the data collection of Fig 1, we would like to highlight the full coverage of diverse phage types highly associated with the model performance.

The feature engineering/extraction step was not well explained in the main part of the manuscript but was emphasized in fig 1. Need to explain it in the results.

Response: We appreciate the reviewer's comment and have moved the methods section before results section for a better manuscript structure (line 118-129).

12X in fig1 was ambiguous. Add some annotation to that.

Response: We appreciate the reviewer's comment and have updated the annotation of 12X transformer blocks in the revised Figure 1.

Better to describe the threshold 1 and 2 in the fig 1 as well. In my understanding, there are 2 steps of classification: step 1 is to find if there is a lysogenic gene in the input sequence; step 2 is a final prediction of phage lifecycles. How does the author integrate the prediction of step 1 to step 2 is ambiguous.

Response: We appreciate the reviewer's comment and have updated threshold 1 and 2 in the revised Figure 1. The aggregation from threshold 1 to threshold 2 were provided in the script (predict_lyso_vs_lytic.py) as below. Basically, the formula calculates the percentage of number of DNA sequences that have high similarity with lysogenic gene markers (>0.9) in the total DNA sequences.

```
p = np.sum(((r[s:e]))>thresh1)/(e-s)
if p >= thresh2:
    out = "Predict: Lysogenic"
else:
    out = "Predict: Lytic"
```

Fig2.

The 2 colors are too similar. A more muted color palette is recommended. And it is more natural to also compare to other similar tools in this figure.

Response: We appreciate the reviewer's comment and have updated Figure 3 (previous Figure 2) in the revised manuscript. Based on the performance of five tools on the test dataset of 374 phage complete genomes, we decided to test only DeepPL and PhaTYP for two case studies.

More analysis of the explainability of DeepPL

Since the authors manually selected lysogenic genes as the input for the model instead of using the whole phage genomes as input, it would be great if the pattern or motif can be identified from

the DNA sequences. Then using the found pattern to enhance the explianability of this model will further refine this work given the fact that DNABERT is multihead attention model and it will emphasize on certain part of the input sequences.

Response: We appreciate the reviewer's comment. As we mentioned in the introduction and method section, temperate phage can display both lytic and lysogenic cycles whereas virulent phage can only display lytic phage. The key genetic difference between these two phage types is the lysogenic genes within the temperate phage genomes (around 8% within the phage complete genome). Therefore, we manually selected lysogenic genes as the input for fine-tuning instead of the whole phage genome, which will increase background noise. One challenge of the fine-tuning process is the requirement of gpu memory. Therefore, we agree with the reviewer that it would be great if the valuable pattern could be identified from the DNA sequences, the lysogenic gene in particular. It will help to reduce the training dataset size and minimize the computing memory requirement. More analysis regarding the pattern extraction has been conducted and will be presented in our future work.
