# Supplementary material for: DeepPL: A deep-learning-based tool for the prediction of bacteriophage lifecycle
Source: PLoS Comput Biol. 2024 Oct 17;20(10):e1012525. doi: 10.1371/journal.pcbi.1012525 (PMC11521287; doi:10.1371/journal.pcbi.1012525)
Supplement: Attachment — Submitted filename: 2nd-review.pdf [file pcbi.1012525.s006.pdf]

The authors have addressed all my questions, resulting in a large enhancement of the text's narrative and providing more clear revised figures, especially Fig 1 and Fig 2. Furthermore, in the revised version the authors well combined the suggestions from reviews and make the text more detailed. The study's distinctiveness is: 1. Develop a fine-tuned DNABERT model DeepPL for lysogenic/lytic bacteriophages prediction. 2. Compare DeepPL with other existing methods to show the best performance. 3. Leverage their exclusive experimental bacteriophages data and simulated metagenome contigs for further validation. Together, DeepPL is a meaningful and powerful tool for prediction lysogenic/lytic bacteriophages. My comments/ questions to the authors in this round mainly refer to the model fine-tuning and small edits in some figures.

1. It will be more beneficial for the readers and potential users to know how exactly the fine-tuned steps are implemented. The following details could be intriguing: 1) Does the original 12 transformer blocks are all frozen, unfrozen or partial frozen during the fine-tuning step? 2) Which type of classification layers are used here? 3) How does the author come up with the threshold 2 = 0.016?

2. For the right panel of Fig. 1, the 12x transformer blocks appeared twice, it could be misleading to think that the fine-tuning step involving another 12x transformer blocks.
