# Supplementary material for: DeepPL: A deep-learning-based tool for the prediction of bacteriophage lifecycle
Source: PLoS Comput Biol. 2024 Oct 17;20(10):e1012525. doi: 10.1371/journal.pcbi.1012525 (PMC11521287; doi:10.1371/journal.pcbi.1012525)
Supplement: Attachment — Submitted filename: Response letter to reviewers VWu.docx [file pcbi.1012525.s007.docx]

**Response Letter to the Reviewer's Comments**

**Manuscript ID**: PCOMPBIOL-D-24-00537

**Manuscript Title:** DeepPL: a deep-learning-based tool for the prediction of bacteriophage lifecycle

Author(s): Yujie Zhang, Mark Mao, Robert Zhang, Yen-Te Liao, and Vivian C.H. Wu

Reviewer #1: The authors addressed all my comments.

Reviewer #2: The authors have addressed my comments and have improved the quality and rigor of their manuscript. However, they still need to include some details in the main text. I don't think that responses that point to github scripts, i.e "The script (batch_process_results.py) provided in the GitHub provided the function of adjusting the setting of threshold 2 and generating the error rate of test dataset." are adequate. Please, include in the paper a simple description of how you choose the value of your parameter. The reader of a journal article is not supposed to parse a github script. The justification of using a larger step size of 91 should also be included in the main text.

*Response: We appreciate the reviewer's comment and have updated the information in the revised manuscript (line 163-165, 171-172).*

Reviewer #3:

The authors have addressed all my questions, resulting in a large enhancement of the text's narrative and providing more clear revised figures, especially Fig 1 and Fig 2. Furthermore, in the revised version the authors well combined the suggestions from reviews and make the text more detailed. The study's distinctiveness is: 1. Develop a fine-tuned DNABERT model DeepPL for lysogenic/lytic bacteriophages prediction. 2. Compare DeepPL with other existing methods to show the best performance. 3. Leverage their exclusive experimental bacteriophages data and simulated metagenome contigs for further validation. Together, DeepPL is a meaningful and powerful tool for prediction lysogenic/lytic bacteriophages. My comments/ questions to the authors in this round mainly refer to the model fine-tuning and small edits in some figures.

1. It will be more beneficial for the readers and potential users to know how exactly the fine-tuned steps are implemented. The following details could be intriguing: 1) Does the original 12 transformer blocks are all frozen, unfrozen or partial frozen during the fine-tuning step? 2) Which type of classification layers are used here? 3) How does the author come up with the threshold 2 = 0.016?

*Response: We appreciate the reviewer's comment, and please refer to the following responses to the corresponding questions.*

1) *Twelve transformer blocks are unfrozen during the fine-tuning process.*
2) *The classification layers are* *binary classification.*
3) *We have updated the information in the revised manuscript (line 171-172).*

2. For the right panel of Fig.1, the 12x transformer blocks appeared twice, it could be misleading to think that the fine-tuning step involving another 12x transformer blocks. *Response: We appreciate the reviewer's comment and have updated Figure 1 in the revised manuscript. We would like to keep 12x transformer blocks to indicate the detailed model structure used for fine turning. Therefore, we changed the color of fine-tuning model, consistent with the color of pre-trained model, to indicate the 12X transformer blocks from pre-training were further used for fine tuning.*
